# The Influence of Phenol on the Growth, Morphology and Cell Division of *Euglena gracilis*

**DOI:** 10.3390/life13081734

**Published:** 2023-08-12

**Authors:** Alexandra Lukáčová, Diana Lihanová, Terézia Beck, Roman Alberty, Dominika Vešelényiová, Juraj Krajčovič, Matej Vesteg

**Affiliations:** 1 Department of Biology, Ecology and Environment, Faculty of Natural Sciences, Matej Bel University, 974 01 Banská Bystrica, Slovakiaterezia.gasparcova@umb.sk (T.B.);; 2 Institute of Biology and Biotechnology, Faculty of Natural Sciences, University of Ss. Cyril and Methodius, 917 01 Trnava, Slovakia

**Keywords:** bleaching, bioindicator, monocyclic aromatic hydrocarbons, lipofuscin, pollutant

## Abstract

Phenol, a monocyclic aromatic hydrocarbon with various commercial uses, is a major pollutant in industrial wastewater. *Euglena gracilis* is a unicellular freshwater flagellate possessing secondary chloroplasts of green algal origin. This protist has been widely used for monitoring the biological effect of various inorganic and organic environmental pollutants, including aromatic hydrocarbons. In this study, we evaluate the influence of different phenol concentrations (3.39 mM, 3.81 mM, 4.23 mM, 4.65 mM, 5.07 mM, 5.49 mM and 5.91 mM) on the growth, morphology and cell division of *E. gracilis*. The cell count continually decreases (*p* < 0.05–0.001) over time with increasing phenol concentration. While phenol treatment does not induce bleaching (permanent loss of photosynthesis), the morphological changes caused by phenol include the formation of spherical (*p* < 0.01–0.001), hypertrophied (*p* < 0.05) and monster cells (*p* < 0.01) and lipofuscin bodies. Phenol also induces an atypical form of cell division of *E. gracilis*, simultaneously producing more than 2 (3–12) viable cells from a single cell. Such atypically dividing cells have a symmetric “star”-like shape. The percentage of atypically dividing cells increases (*p* < 0.05) with increasing phenol concentration. Our findings suggest that *E. gracilis* can be used as bioindicator of phenol contamination in freshwater habitats and wastewater.

## 1. Introduction

*Euglena gracilis* is a model euglenoid flagellate (unicellular alga, protist) frequently occurring in freshwater habitats. It belongs to euglenids, which together with diplonemids, symbiontids and kinetoplastids (trypanosomatids and bodonids) belong to the protist phylum Euglenozoa [1] and the protist group Discoba formerly classified within the eukaryotic supergroup Excavata [2]. Wild-type strains of *E. gracilis* (e.g., strains *Z* and *bacillaris*) can live as photolithoautotrophs since they possess complex green chloroplasts bounded by three membranes (secondary chloroplasts) descending from a chlorophycean algal symbiont related to the genus *Pyramimonas* already utilizing primary chloroplasts of cyanobacterial origin for photosynthesis [3,4]. All *E. gracilis* strains including “white mutants” (see below) can feed chemoorganoheterotrophically (osmotrophically) utilizing various organic carbon sources available in the environment such as glucose, lactate or ethanol. *E. gracilis* wild-type strains, however, usually combine both these nutrition modes, being mixotrophs [5,6,7]. These flagellates are able to tolerate various changes in environmental conditions including a wide range of pH (3–9), temperature, oxygen concentration, salinity and also ionizing irradiation [8,9].

*E. gracilis* has many biotechnological implications for the production of pharmaceuticals, dietary supplements, biomaterials and biofuels [10,11,12,13,14]. It is a natural source of bioproducts such as dietary proteins containing all essential amino acids, wax esters (a feedstock for biofuels), polyunsaturated fatty acids and its storage polysaccharide paramylon (a β-1,3-glucan) with immunostimulatory properties and broad applications in medicine [10,11,12,14]. Although *E. gracilis* can also produce large amounts of provitamin A (β-carotene) and vitamins C (ascorbate) and E (α-tocopherol), it is not able to synthesize thiamine (vitamin B_1_) and cobalamin (vitamin B_12_) and, therefore, these two vitamins must be added to media for the growth of its axenic cultures. The high price of these vitamins is the highest limitation factor for obtaining high yields of *E. gracilis* biomass for biotechnological purposes. On the other hand, no addition of vitamins is required when *E. gracilis* is grown in co-culture with the bacteria *Pseudobacillus badius* and *Lysinibacillus boronitolerans* and with the micromycete *Cladosporium westerdijkiae*, suggesting that these microorganisms can produce sufficient amounts of vitamins B_1_ and B_12_ for this flagellate [13,14]. Many *E. gracilis* cells in this co-culture are attached to the fungal hyphae by flagella, suggesting that such bioflocculation offers the possibility for effective harvesting of *E. gracilis* biomass simply using nets [13,14].

*E. gracilis* is also a suitable model microorganism for monitoring the biological effects of various environmental pollutants and ecotoxicological risk assessment, as well as for bioremediation of polluted water [14]. It has been used to test the biological effect of heavy metals such as cadmium, copper, chromium [15,16] and nickel [17] as well as monocyclic aromatic hydrocarbons (arenes) such as benzene, toluene, ethylbenzene, xylenes and their mixture—BTEX [18,19]. The *E. gracilis* chloroplast genome of cyanobacterial origin is sensitive to various antibacterial agents that do not affect eukaryotic nuclear genetic apparatus [20,21,22]. The growth of *E. gracilis* in the presence of compounds inhibiting bacterial replication, transcription and translation leads to the permanent loss of the ability to photosynthesize—“bleaching” [20,21,22]. For example, streptomycin and ofloxacin specifically inhibit protein synthesis on plastid ribosomes and plastid DNA replication in *E. gracilis*, respectively. The bleaching process is accompanied by selective loss of plastid-encoded genes, while it has no effect on *E. gracilis* cell growth and viability under laboratory conditions [20,21,22]. Various stable *E. gracilis* bleached “white” mutant strains were produced that did not even possess an identifiable plastid remnant [5,6,7,20,21,22].

In contrast to antibacterial agents, the most common responses of *E. gracilis* to monocyclic aromatic hydrocarbons include irregular cell shape, hypertrophy and intracellular formation of lipofuscin granules (bodies), but not bleaching [18,19]. The production of lipofuscin was also observed in mammalian and other animal cells as a response to various environmental factors, aging and stress conditions [23,24]. Analytical methods recommended for the determination of monocyclic aromatic hydrocarbons in surface and groundwater include headspace gas chromatography (headspace GC), gas chromatography with mass spectrometry (GC-MS) and gas chromatography with flame ionization detector (GC-FID). Although these techniques are reliable, they are complicated, time-consuming and quite expensive [18]. These disadvantages could be overcome by using a suitable bioindicator of organic contamination by arenes. Due to its short generation time and the highly specific and rapid biological responses of *E. gracilis* to the presence of arenes in the environment, it seems to be an appropriate candidate for this purpose.

Among arenes (aromatic hydrocarbons), one of the most common organic water pollutants is also phenol (hydroxybenzene). There are many different sources of environmental contamination by phenol including waste from paper manufacturing, the pharmaceutic and petrochemical industries, agriculture, coal processing or even municipal wastes [25]. Phenol has also been used very often in molecular biological laboratories as the basic substance for DNA and RNA isolation. Phenol has been repeatedly detected in wastewater from various industrial sources with concentration ranges of 1–10,000 mg/L [26,27,28], while the ingestion of 1 g of phenol is detrimental to human life [29]. However, depending on species, it can be toxic at a concentration of 5 mg/L [30]. Phenol and related compounds have been thus considered as priority pollutants by the European Union (EU) and the United States Environmental Protection Agency (USEPA) [31]. Since many industrial employees and researchers have been frequently exposed to phenol, the studying of its biological effects on various organisms and monitoring its presence in the environment should be of special interest.

In this study, we decided to evaluate the influence of phenol on *E. gracilis*. The key goals of our study were as follows: (1) to experimentally determine the concentrations of phenol (sublethal range of phenol concentrations) with observable biological effects on the growth, morphology and cell division of *E. gracilis*, but not completely inhibiting the cell growth, (2) to describe the altered characteristics caused by phenol treatment and (3) to monitor and compare the influence of various sublethal doses of phenol on *E. gracilis* over time (1 h, 24 h, 72 h, 7 days, 10 days and 2 weeks of cultivation).

## 2. Material and Methods

### 2.1. Cultivation Conditions, Phenol Treatment and Monitored Parameters

*Euglena gracilis* strain *Z* (Pringsheim strain *Z*, SAG 1224-5/25 Collection of Algae, Göttingen, Germany) was used in this study to monitor the effect of phenol. Approximately 1.75 × 10^6^ cells were diluted in Erlenmeyer flasks containing 35 mL of liquid Cramer and Myers (CM) medium (not containing agar) [32] supplemented with ethanol (final concentration 0.8%) and pH adjusted to 6.9 with 0.1 M NaOH [33], resulting in a final density of 5 × 10^4^ cells per ml. The concentrations of vitamins B_1_ and B_12_ were the same as described in Cramer and Myers [32]. The treatment was performed in closed 1.5 mL Eppendorf tubes containing 1 mL (5 × 10^4^ cells) of stock axenic culture.

Next, 100 mL of phenol stock solution containing 8 g of phenol was freshly prepared (final concentration 0.85 M, 80 mg/mL). Then, 4 µL, 4.5 µL, 5 µL, 5.5 µL, 6 µL, 6.5 µL and 7 µL of phenol stock solution were added to Eppendorf tubes containing 1 mL (5 × 10^4^ cells) of *E. gracilis* axenic culture resulting in final concentrations of 3.39 mM (318.73 µg/L, 3.81 mM (358.38 µg/L), 4.23 mM (398.01 µg/L), 4.65 mM (437.59 µg/L), 5.07 mM (477.14 µg/L), 5.49 mM (516.64 µg/L) and 5.91 mM (556.11 µg/L), respectively. All experiments were performed in triplicate. Untreated controls (with no phenol added) were also grown in triplicate.

Untreated controls and treated samples were grown under LED illumination (3360 lm) at 25 °C with a 16 h light and 8 h dark cycle. The cell growth (cell density), atypical cell division (producing more than two cells from a single cell), the presence of lipofuscin bodies and other morphological changes such as the presence of hypertrophied and monster cells (gigantic cell with abnormal shape) were monitored after 1 h, 24 h, 72 h, 7 days, 10 days and 2 weeks of cultivation. The cell density, the percentage of atypically dividing cells and the percentage of cells with lipofuscin bodies, monster cells and hypertrophied cells were determined by direct counting in a Bürker chamber.

### 2.2. Light Microscopy

Controls and treated samples were observed under a Panthera L Binocular Microscope with Color Corrected Infinity Optical System (Motic, Hong Kong). Images were processed in Panthera App version 2.1.3 (Motic, Hong Kong). Light microscopy was used for capturing the images of non-dividing cells with normal size and shape, normally dividing cells, atypically dividing cells, non-motile spherical cells, hypertrophied cells, monster cells and cells with lipofuscin bodies. In these cases, no fixation of cells was used and 20 µL of culture was added to the slide. Light microscopy was used also for the counting of cells and cells with the altered characteristics in the Bürker chamber. For this purpose, 2 µL methylene blue and 10 µL formaldehyde were added to 20 µL of phenol-treated (as well as untreated control) cultures and water (68 µL) was added, resulting in a final sample volume of 100 µL, which was added to the Bürker chamber.

### 2.3. Transmission Electron Microscopy (TEM)

Transmission electron microscopy images of *E. gracilis* ultrastructure of the nucleus and nucleolus in the control and treated cultures were obtained using a Jeol JEM 2100-Plus microscope at 120 kV. Cells were fixed for 2 h with 2.5% glutaraldehyde in 0.1 M PHEM buffer. After washing, the cells were embedded in 2% low-melting-agarose-coated coverslips and post-fixed with 1% osmium tetroxide for 1 h at 4 °C. Acetone dehydration was followed by embedding in EmBed812. Polymerized resin blocks were ultra-thinly sectioned and stained with 1% uranyl acetate and 2% lead citrate. All chemicals were purchased from Electron Microscopy Science, USA. TEM was used to observe atypically dividing cells and their dividing nuclei to study the nature of atypical cell and nuclear division.

### 2.4. Confocal Microscopy of Cells with Atypical Cell Division

Transmitted light and confocal images of *E. gracilis* cells exhibiting atypical cell division were recorded on a Nikon/Yokogawa CSU-W1 spinning disk confocal microscope with a 100×/1.4 N.A. oil-immersion objective (CFI Plan Apo VC 100X Oil) and back-illuminated sCMOS camera (PRIME BSI, Teledyne Photometrics). Fluorescence confocal images of Hoechst-stained nuclei [34] were recorded using 405 nm laser excitation and a 430–480 nm band-pass emission filter. Z-stacks were recorded with 100 ms exposure time per plane and 300 nm z-steps. Confocal microscopy was used to study the synchronization of the atypical cell and nuclear division.

### 2.5. Statistical Analysis

All statistical analyses were performed using the IBM SPSS software, version 28.0 (IBM Corp., Chicago, IL, USA). The limit for the statistical significance was set to *p* < 0.05. The means and standard errors were calculated from triplicate results. Percentage changes D% = (T_x_ − T_0_)/T_0_ × 100 related to the untreated control were calculated and compared between groups. The influence of phenol treatment was assessed using a two-way (cultivation time × phenol treatment) ANOVA with repeated measures. Variance homogeneity between examined groups was determined using Levene’s test. The effect size of the main independent factors (cultivation time and phenol treatment), as well as their interaction effect (cultivation time × phenol treatment) on dependent variables (morphological cell characteristics), was estimated as Cohen´s d. After testing of the significance of differences through ANOVA, a post hoc Bonferroni test was used to evaluate significant differences in the pairwise comparisons.

## 3. Results

### 3.1. The Effect of Phenol on E. gracilis Cell Growth

**Figure 1:**
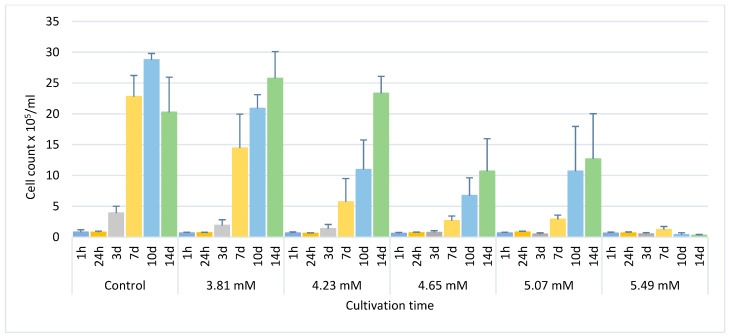
The influence of phenol on the growth of *E. gracilis*. Control—untreated control. h—hour, d—day. Columns represent means (cell count × 10^5^/mL) and bars standard errors calculated from triplicate results. Within-group differences: control 1 h vs. 10 d and control 24 h vs. 10 d, *p* < 0.05. Between-group differences: control vs. 4.65 mM and control vs. 5.07 mM, *p* < 0.05; 3.81 mM vs. 5.49 mM, *p* < 0.01 and control vs. 5.49 mM, *p* < 0.001.

The control untreated *E. gracilis* cultures reached a mean cell count of 22.8 × 10^5^ cells/mL after 7 days (exponential growth phase), which was consistent with previous studies [21]. After 10 days and 14 days, the mean cell counts of cells in control culture were ~28.8 × 10^5^ cells/mL and 20.3 × 10^5^ cells/mL, respectively (Figure 1). These experiments have confirmed that after one week, the untreated *E. gracilis* culture is generally in the exponential phase of growth, and within the next week, it is generally in the stationary phase.

In all treated cultures, the growth rate was significantly lower (*p* < 0.05–0.001) than in control culture and it decreased with increasing phenol concentration (Figure 1). In cultures treated with 3.81 mM and 4.23 mM phenol, the mean count of cells per ml after 7 days was 14.5 × 10^5^ and 5.7 × 10^5^, respectively, while the maximum cell number was reached only after 14 days in both these treated cultures: 25.7 × 10^5^/mL for 3.81 mM and 23.3 × 10^5^/mL for 4.23 mM (Figure 1). In the cultures treated with 4.65 mM and 5.07 mM phenol, the maximum number of cells per mL was observed after 10 and 14 days (range: 6.7–10.7 × 10^5^ and 10.7–12.7 × 10^5^/mL, respectively). The maximum cell count of cells in the culture treated with 5.49 mM phenol, only 1.24 × 10^5^ cell/mL, was reached after 7 days. Almost no cells survived after 14 days of treatment with 5.49 mM phenol (Figure 1). No cells survived after treatment with 5.91 mM phenol (and higher phenol concentrations), while treatment with 3.39 mM phenol (and lower phenol concentrations) had no effect on cell growth, morphology or cell division in comparison with the control. Therefore, the results for concentrations 3.39 mM (and lower) and 5.91 mM (and higher) are not presented and were omitted from final analysis. The sublethal range of phenol concentrations for *E. gracilis* has thus been estimated to be 3.81–5.49 mM.

### 3.2. The Effect of Phenol on the Morphology of E. gracilis

**Figure 2:**
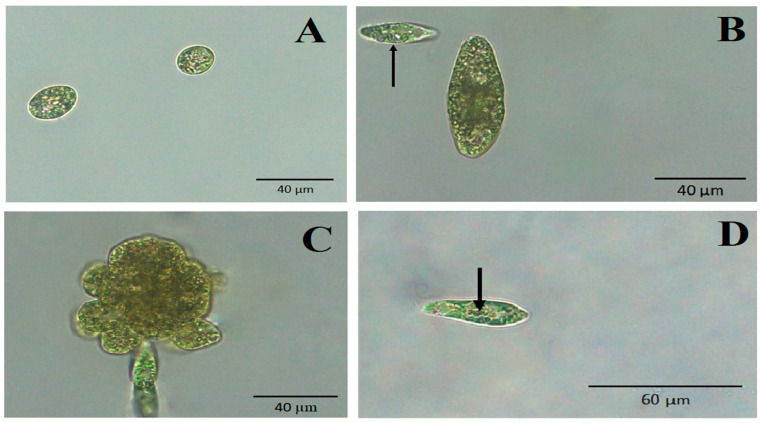
Morphological changes of *E. gracilis* induced by phenol. (**A**) Non-motile spherical cells (4.65 mM phenol treatment for 24 h). (**B**) Hypertrophied cell, arrow—cell with normal size (5.49 mM phenol treatment for 10 days). (**C**) Monster cell (gigantic cell with abnormal shape) (5.49 mM phenol treatment for 7 days). (**D**) Cell with lipofuscin bodies (arrow) (3.81 mM phenol treatment for 14 days). Images were captured using a Panthera L Binocular Microscope with Color Corrected Infinity Optical System (Motic, Hong Kong) and processed in Panthera App version 2.1.3 (Motic, Hong Kong). No fixation of samples was used.

The monitored changes in *E. gracilis* morphology after phenol treatment included cells with spherical shape (Figure 2A), hypertrophied cells (Figure 2B), monster cells (gigantic cells with abnormal shape) (Figure 2C) and cells containing intracellular lipofuscin bodies (Figure 2D). In untreated control cultures, no such morphological changes were observed and in cultures treated with 3.39 mM phenol, cells with the mentioned morphological changes were very rare and almost uncountable.

**Figure 3:**
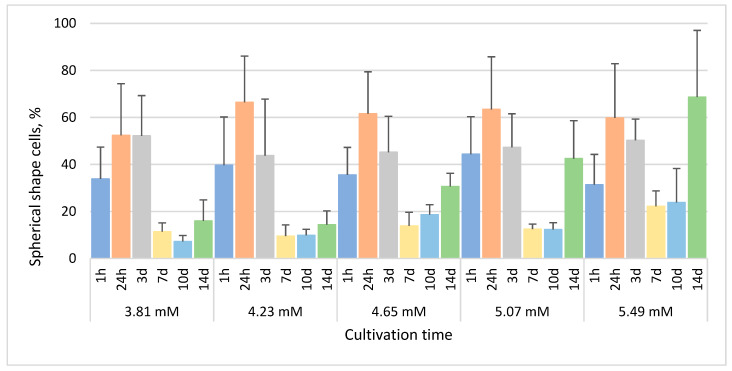
The percentage of *E. gracilis* non-motile cells with spherical shape after phenol treatment. h—hour, d—day. Columns represent means (%) and bars standard errors calculated from triplicate results. No statistically significant differences (*p* < 0.05) within or between groups were found.

Spherical cells were observed even after 1 h in all phenol-treated cultures, comprising a mean 31.4–44.4% of all cells. In the culture treated with 3.81 mM phenol, the highest mean percentage (~52%) was observed after both 24 h and 3 days, and the lowest mean percentage (7.2%) after 10 days (Figure 3). In the cultures treated with 4.23 mM, 4.65 mM, 5.07 mM and 5.49 mM phenol, the maximum percentage of cells with spherical shape among triplicates (87–96%) was observed after 24 h, and then their percentage decreased during the next 6 days. The lowest percentage of spherical cells among triplicates (0–12%) was observed after 7 and 10 days in all treated cultures, while their percentage increased approximately 2-fold (4–24%) 14 days after phenol treatment.

**Figure 4:**
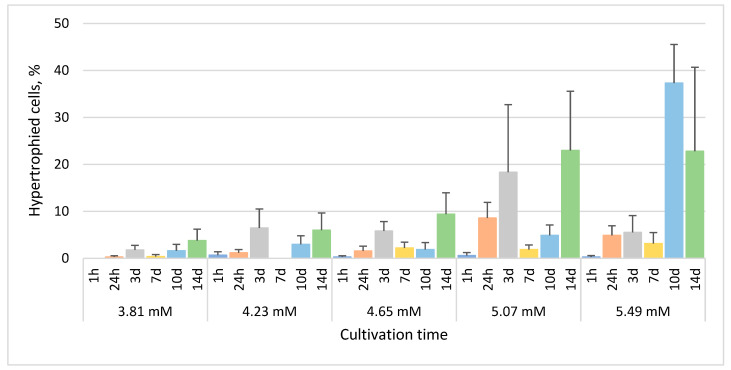
The percentage of *E. gracilis* hypertrophied cells after phenol treatment. h—hour, d—day. Columns represent means (%) and bars standard errors calculated from triplicate results. No statistically significant differences (*p* < 0.05) within or between groups were found.

The mean percentage of hypertrophied cells generally increased with increasing concentration of phenol (Figure 4). The maximum percentages of hypertrophied cells among triplicate cultures treated with 3.81mM, 4.23 mM, 4.65 mM, 5.07 mM and 5.49 mM phenol after 14 days were 8.4%, 12.9%, 14.4%, 43.5% and 62.5%, respectively. However, the maximum mean percentage of these cells (37.3%) was observed after just 10 days in the culture treated with 5.49 mM phenol (Figure 4).

**Figure 5:**
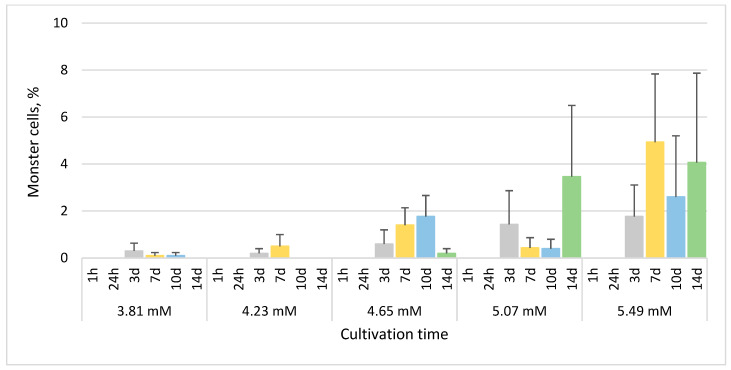
The percentage of *E. gracilis* monster cells after phenol treatment. h—hour, d—day. Columns represent means (%) and bars standard errors calculated from triplicate results. No statistically significant (*p* < 0.05) within-group differences were found. Between-group differences: 5.07 mM vs. 5.49 mM, *p* < 0.05; 3.81 mM vs. 5.49 mM, 4.23 mM vs. 5.49 mM, and 4.65 mM vs. 5.49 mM, *p* < 0.001.

The maximum mean percentages of monster cells generally increased with increasing concentration of phenol. No monster cells were observed after 1 h and 24 h in all treated cultures, and the mean percentage of monster cells was also zero or close to zero after 14 days in cultures treated with 3.81 mM, 4.23 mM and 4.65 mM phenol (Figure 5). The highest mean percentages of monster cells were observed in the culture treated with 5.49 mM phenol after 7 days (4.9%) and 14 days (4.1%) (Figure 5).

**Figure 6:**
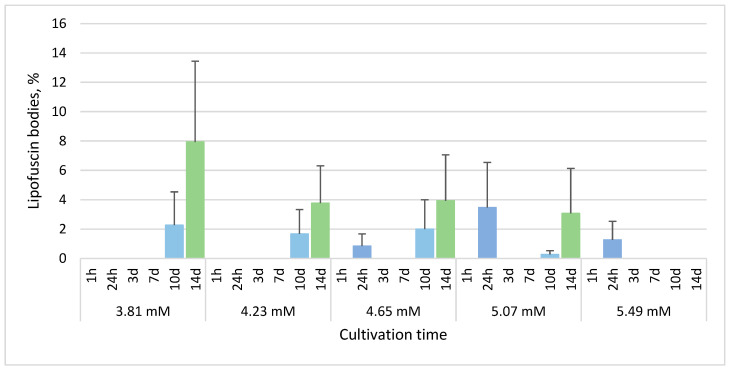
The percentage of *E. gracilis* cells containing lipofuscin bodies after phenol treatment. h—hour, d—day. Columns represent means (%) and bars standard errors calculated from triplicate results. No statistically significant differences (*p* < 0.05) within or between groups were found.

Intracellular lipofuscin bodies began to form in the cultures treated with 3.81 mM, 4.23 mM, 4.65 mM and 5.07 mM phenol mainly after 10 days of treatment, with the maximum mean percentages of cells containing these bodies after 14 days reaching 7.9%, 3.8%, 3.9% and 3.1%, respectively (Figure 6). In the culture treated with 5.49 mM phenol, cells containing lipofuscin bodies (mean 1.3%) were observed only after 24 h (Figure 6).

### 3.3. Phenol Induces Atypical Cell Division of E. gracilis

**Figure 7:**
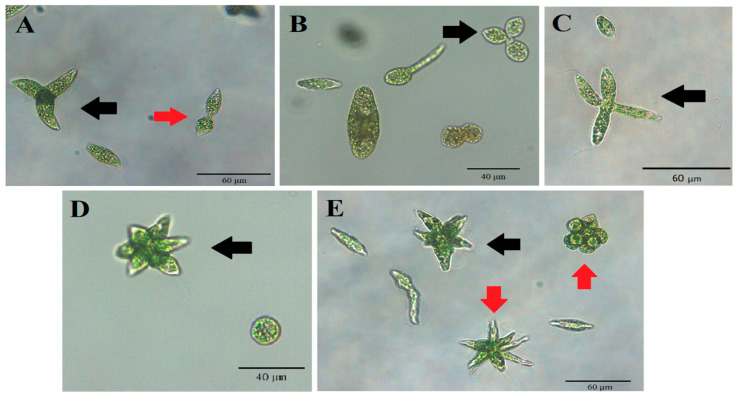
The forms of atypical cell division of *E. gracilis*. (**A**) Normal cell division (red arrow), atypical cell division (black arrow) and non-dividing cell (no arrow). (**B**) Three daughter cells arising from one mother cell (black arrow). (**C**) Four daughter cells arising from one mother cell (black arrow). (**D**) Seven daughter cells arising from one mother cell (black arrow). (**E**) Seven (black arrow) and eight daughter cells (red arrows) arising from one mother cell. Images were captured using a Panthera L Binocular Microscope with Color Corrected Infinity Optical System (Motic, Hong Kong) and processed in Panthera App version 2.1.3 (Motic, Hong Kong). No fixation of samples was used. These images were captured after 5.49 mM phenol treatment for 10 days.

**Figure 8:**
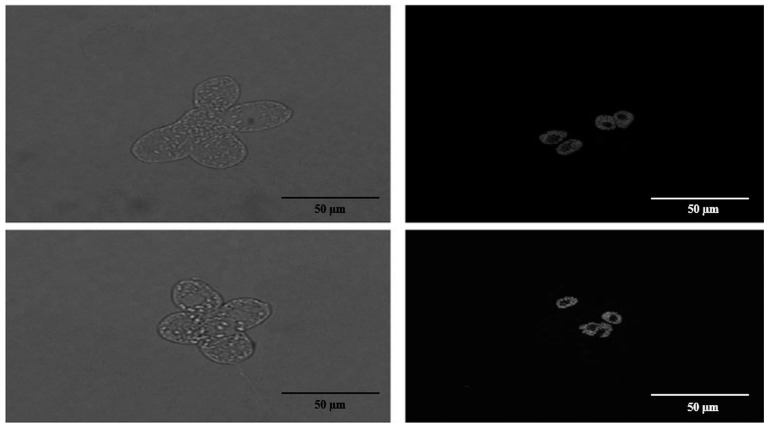
Confocal microscopy of *E. gracilis* cell containing four nuclei simultaneously dividing into four cells. This image was captured after 5.49 mM phenol treatment for 10 days.

**Figure 9:**
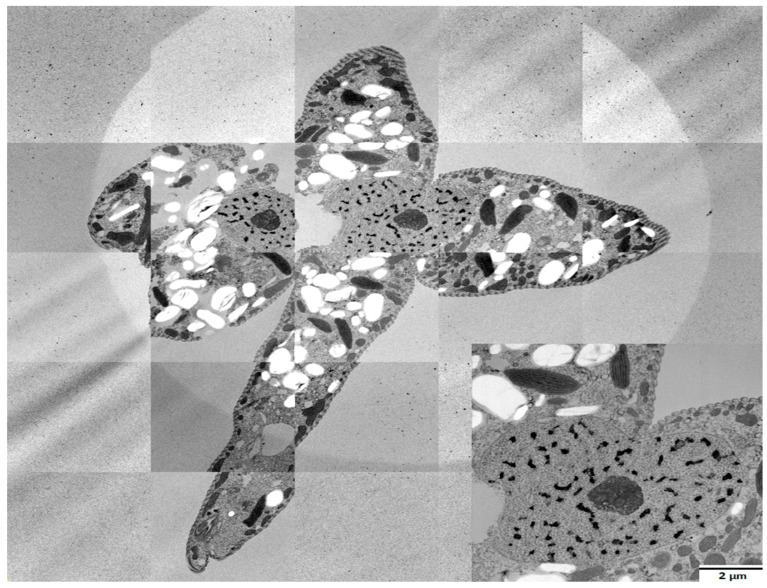
Transmission electron microscopy of *E. gracilis* dividing into four cells. This image was captured after 5.49 mM phenol treatment for 10 days, and it was created by tagging together various micrographs. The detail of the dividing nucleus is shown in the lower right corner.

We have observed an atypical form of cell division of *E. gracilis* cells in all cultures treated with all selected phenol concentrations. In contrast to standard longitudinal cell division producing two cells, during this atypical cell division, three, four, five, six, seven, eight or even more cells arise from a single *E. gracilis* cell. Each of these arising cells have a visible flagellum at the anterior end and stigma during division (Figure 7). The results from confocal microscopy suggest that the number of nuclei in an atypically dividing cell is the same as the number of forming daughter cells (Figure 8). An electron microscopy image of *E. gracilis* dividing into four cells and its dividing nucleus is shown in Figure 9. These atypically dividing cells are symmetric, viable and motile. We have also observed multiple viable dividing cells attached only by very distal parts of their posterior ends, suggesting that they most likely normally finish the cell division.

**Figure 10:**
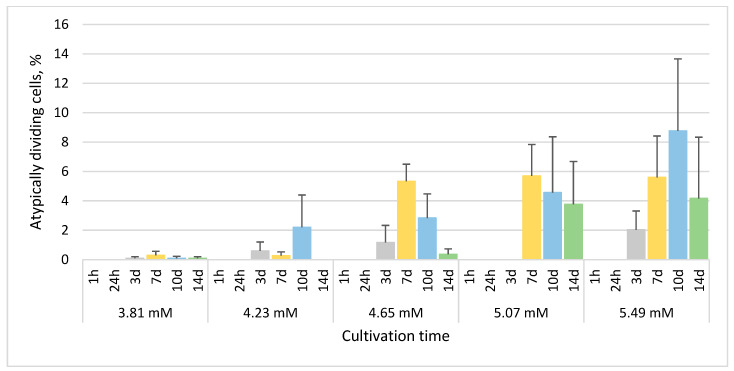
The percentage of *E. gracilis* atypically dividing cells after phenol treatment. h—hour, d—day. Columns represent means (%) and bars standard errors calculated from triplicate results. No statistically significant differences (*p* < 0.05) within groups were found. Between-group difference: 3.81 mM vs. 5.49 mM, *p* < 0.05.

No atypically dividing cells were observed in the culture treated with 3.39 mM phenol. The most of atypically dividing cells were observed after 7 and/or 10 days in all treated cultures (except for the culture treated with 3.39 mM phenol). The mean percentages of atypically dividing cells generally increased with increasing concentration of phenol with the maximum mean (8.8%) in the culture treated with 5.49 mM phenol after 10 days (Figure 10).

### 3.4. Statistical Analysis

The cell count statistically significantly continually decreased (from −8.2 × 10^5^ to −12.3 × 10^5^/mL, *p* < 0.05–0.001) with the increasing phenol concentration over time. The statistically significant morphological changes caused by phenol in comparison with the untreated control include the formation of spherical (from +28.8 to +42.7%, *p* < 0.01–0.001), hypertrophied (+12.3%, *p* < 0.05), monster (+2.2%, *p* < 0.01) and atypically dividing cells (+3.4%, *p* < 0.05). However, the increased occurrence of lipofuscin bodies after phenol treatment was not statistically significant.

Two-way ANOVA by means of repeated measures verified the influence of cultivation period (factor: time) and phenol concentration (factor: treatment) on the growth rate, morphology and cell division of *E. gracilis* (Supplementary Tables S1–S3). The main effect analysis revealed two large (Cohen′s d ≅ 0.8, *p* < 0.001) universal and equal main effects (time and treatment) for several monitored parameters. Specifically, a significant effect of time on cell count and formation of spherical shape cells was found, F_(2, 30)_ = 38.160, *p* < 0.001 and F_(2, 23)_ = 7.064, *p* = 0.004, respectively. For both these parameters, the main effect of treatment also showed the significant influence, F_(5, 12)_ = 9.024 and F_(5, 12)_ = 15.748, *p* < 0.001 for both. For most of the other monitored cell characteristics (hypertrophied cells, monster cells and atypical cell division), even with statistically significant differences (*p* < 0.05–0.01) occurring over time and treatments, the impact of these main effects was relatively small to moderate (d = 0.2–0.7). In addition, no significant interaction effect (time × treatment) could be demonstrated for all monitored cell characteristics, except for the cell count (F_(12, 30)_ = 4.146, *p* < 0.001).

## 4. Discussion

*Euglena gracilis* has been widely used as a model flagellate to monitor the influence of various chemical as well as physical factors. While some factors such as UV, high temperature and most antibiotics cause permanent irreversible loss of photosynthetic ability (bleaching) [5,6,7,20,21,22], the typical responses of *E. gracilis* to benzene, toluene and xylene occurring within 24 h include irregular shape, hypertrophy, intracellular formation of lipofuscin granules (bodies) and decreased chlorophyll content [18,19], but not permanent bleaching connected with the loss of plastid genes. In this study, we have confirmed that phenol has a similar effect on *E. gracilis* to other monocyclic aromatic hydrocarbons and that it does not induce bleaching. In addition to the responses of *E. gracilis* observed after treatment with other arenes, phenol can also induce atypical cell division, producing more than two cells from a single cell.

We have monitored the short- (1 h, 24 h) as well as long-term effect (3, 7, 10 and 14 days) of phenol on *E. gracilis*. The short-term (1 h) influence on another closely related euglenid species, *Euglena agilis* Carter, includes reduced photosynthetic rate and motility, and triggers change in the swimming velocity [35]. However, such rapid phenol toxicity testing does not allow monitoring of the growth rate and characteristic morphological changes induced by sublethal doses of phenol over longer time periods. We have experimentally determined that the long-term effect of phenol on *E. gracilis* can be tested using the phenol concentrations 3.81 mM (358.38 µg/L), 4.23 mM (398.01 µg/L), 4.65 mM (437.59 µg/L), 5.07 mM (477.14 µg/L) and 5.49 mM (516.64 µg/L).

Phenol aqueous solutions (1% to 2%) are still frequently used in some countries as a “safe” disinfectant, occasionally causing skin irritation [29], although the toxicity for aquatic animals and humans is within the range 9–25 mg/L [36]. While the phenol concentration in seawater is only up to 130 µg/L, even in polluted fishing areas, it can rapidly rise after uncontrolled release from industry [37]. However, phenol contamination represents a similar or even higher environmental risk for freshwater ecosystems. *E. gracilis*, a freshwater protist/alga highly resistant to various pollutants, is approximately 50-fold less resistant to phenol than metazoans. This should be stressed, while allowing the contamination of natural habitats by phenol, because many other microorganisms need not survive the doses tolerated by metazoan models. Ignoring the limit of resistance of aquatic microbiota against aromatic hydrocarbons could, in turn, lead to the devastation of local and even global water ecosystems.

The growth rate of *E. gracilis* in our experimental sublethal range of phenol concentrations, from 3.81 mM (358.38 µg/L) to 5.49 mM (516.64 µg/L), generally decreased with increasing concentration of phenol (Figure 1). No bleached cells were observed after treatment with any selected phenol concentrations over any selected time periods. The characteristic morphological changes induced by phenol include spherical non-motile cells, hypertrophied and monster cells and the formation of lipofuscin bodies (Figure 2). Hypertrophied cells and monster cells are also formed after cadmium treatment, likely due to the suppression of cytokinesis [38]. The first morphological change induced by phenol includes the formation of spherical cells, which occur already after 1h of phenol treatment, generally reaching the maximum after 24 h (Figure 3). Monster cells occur rather chaotically after 3–14 days of phenol treatment, depending on phenol concentration (Figure 5). The two other morphological changes, formation of hypertrophied cells and cells with lipofuscin bodies, are mainly observable rather later, with the maximum mean percentage of these cells occurring generally after 14 days of phenol treatment in most cases (Figure 4 and Figure 6).

Lipofuscin (“ceroid” or “age pigment”) accumulation has been observed during the senescence of many invertebrate as well as vertebrate cells, including human ones, and its formation is also related to many pathological processes, oxidative stress and the influence of various pollutants [24,25,39]. Lipofuscin bodies are lysosome-derived membrane-bounded structures filled with autofluorescent material (lipofuscin) containing mainly lipids (30–70%), especially oxidized polyunsaturated fatty acids, but also oxidized proteins (20–50%) and metals (mainly Al, Ca, Cu, Fe, K, Mg, Mn, Na and Zn) [39,40]. The formation of cellular structures, which are most likely homologous to lipofuscin bodies—“reddish globules” and “accumulation bodies”—has also been observed in eustigmatophytes and dinoflagellates, respectively [40,41,42]. Based on the presence of lipofuscin bodies also in many other distantly related eukaryotic clades such as euglenids, haptophytes, chromerids, xanthophytes and green algae, and based on common chemical and physical properties of all of these structures, Pilátová et al. [40] have recently suggested that they might be a universal marker of senescence and oxidative stress in all eukaryotes. It is, however, currently unclear if they can be virtually formed in cells of all eukaryotic species. Nevertheless, the common ancestry of all eukaryotes [43,44] and the ability to form lipofuscin bodies in a wide range of distantly related eukaryotic clades [40] favor the hypothesis of their presence as the sign of aging and oxidative stress in the last common eukaryotic ancestor.

The most interesting response of *E. gracilis* to phenol treatment is the atypical cell division. *E. gracilis* reproduces asexually through closed mitosis without the dissolution of the nuclear envelope, without the dissolution of the centrally positioned nucleolus and through longitudinal binary fission starting in the ampula (reservoir), while chromosomes are condensed throughout the whole cell cycle [45]. Sexual reproduction and meiosis are unknown in this flagellate. However, various authors have observed an atypical cell division of a single cell into more than 2 daughter cells (3–12) that are connected by their posterior ends, forming characteristic symmetric “star”-like structures. Such a form of cell division has been observed after the treatment of *E. gracilis* with coumarin [46] and with hexavalent chromium [47] as well as in several-week-old cultures of *E. gracilis* [48]. Subsequently, this kind of cell division was also observed in several-week-old cultures of other *Euglena* spp. including *E. geniculata* Duj., *E. deses* Ehr. (strain 1224-l9a), *E. anabaena* var. *minor* Mainx (strain 1224-2) and *E. viridis* Ehr. (strain 1224-l7b) [49,50]. Zakryś [48,49,50] has suggested that most of these “star”-like dividing cells likely finish cell division, producing normal viable progeny. The transfer of single *E. gracilis* cells dividing into three or four daughter cells into fresh media resulted in normally developing cultures [48]. This is consistent with our observations after phenol treatment, since these “star”-like cells are viable and motile, most of them (if not all) likely have the same number of nuclei (or forming nuclei) as the number of forming cells (Figure 7, Figure 8 and Figure 9), and we have also observed dividing cells forming symmetric “stars” attached only by the most distal parts of their posterior ends. The percentage of these atypically dividing cells increased with increasing concentration of phenol (Figure 10). This atypical cell division is unlikely to be meiosis, because this division displays virtually the same features whether an even or odd number of cells are arising. It seems to be essentially similar to normal longitudinal binary division, having the karyokinesis and cytokinesis synchronized. The treatment of *E. gracilis* with phenol provides a useful tool for studying the nature of this atypical form of cell division in more detail in the future.

## 5. Conclusions

Phenol is a monocyclic aromatic hydrocarbon commonly used in molecular biological laboratories. This arene has broad commercial applications and it is also one of the major organic pollutants in industrial wastewater. *E. gracilis* is a suitable model eukaryotic microorganism for studying the biological effect of various environmental pollutants such as heavy metals and arenes, including phenol. Phenol induces an atypical form of cell division of *E. gracilis,* producing more than two cells from one mother cell, the formation of spherical, hypertrophied and monster cells and cells containing lipofuscin bodies, but it does not cause permanent loss of photosynthesis (bleaching). To our knowledge, this is the first study of the influence of phenol on *E. gracilis* and the first report showing that the atypical cell division of *E. gracilis* can be induced by phenol. Our study may have broad implications in ecotoxicology, since reduced cell count and the presence of *E. gracilis* cells with altered morphology and cell division in freshwater habitats and wastewater can be indicative of phenol contamination. Moreover, the induction of atypical cell division producing more than two cells from a single cell by phenol provides an opportunity for more detailed description and study of the nature of this enigmatic cell biological process.

## Data Availability

Data sharing is not applicable to this article as no datasets were generated or analyzed during the current study.

## Supplementary Materials

The following supporting information can be downloaded at: https://www.mdpi.com/article/10.3390/life13081734/s1, Table S1: Two-way ANOVA: Tests of within-subjects and between-subjects effects for cell count, spherical shape cells, hypertrophied cells, monster cells, lipofuscin bodies and atypically dividing cells of *Euglena gracilis*; Table S2: Two-way ANOVA: Test of pairwise differences (factor: phenol treatment) for cell count, spherical shape cells, hypertrophied cells, monster cells, lipofuscin bodies and atypically dividing cells of *Euglena gracilis*; Table S3: Two-way ANOVA: Test of pairwise differences (factor: time) for cell count, spherical shape cells, hypertrophied cells, monster cells, lipofuscin bodies and atypically dividing cells of *Euglena gracilis*.
