# Supplementary material for: The Influence of Phenol on the Growth, Morphology and Cell Division of Euglena gracilis
Source: Life (Basel). 2023 Aug 12;13(8):1734. doi: 10.3390/life13081734 (PMC10455851; doi:10.3390/life13081734)
Supplement: Supplementary file 1 [file life-13-01734-s001.zip › life-2468922-supplementary.pdf]

The influence of phenol on the growth, morphology and cell division of *Euglena gracilis*

SUPPLEMENTARY MATERIALS

Citation: Lukáčová, A.; Lihanová, D.; Beck, T.; Alberty, R.; Vešelényiová, D.; Krajčovič, J.; Vesteg, M. The Influence of Phenol on the Growth, Morphology and Cell Division of *Euglena gracilis*. *Life* **2023**, *13*, 1734. https://doi.org/10.3390/life13081734

Table S1. Two-way ANOVA: Tests of within-subjects and between-subjects effects for cell count, spherical shape cells, hypertrophied cells, monster cells, lipofuscin bodies and atypically dividing cells of *Euglena gracilis.*

Outcomes	F	df	Sig.	Cohen's d
Main effect – cultivation time × phenol treatment				
Cell count	4.146	12.328, 29.587	<0.001	0.633
Spherical shape cells	0.645	9.685, 23.245	0.757	0.212
Hypertrophied cells	1.686	9.667, 23.200	0.146	0.413
Monster cells	0.634	8.842, 21.222	0.754	0.209
Lipofuscin bodies	0.889	7.612, 18.265	0.541	0.270
Atypically dividing cells	0.865	11.060, 26.544	0.583	0.265
Main effect – cultivation time				
Cell count	38.160	2.466, 29.587	<0.001	0.761
Spherical shape cells	7.064	1.937, 23.245	0.004	0.371
Hypertrophied cells	4.072	1.933, 23.200	0.032	0.253
Monster cells	1.322	1.768, 21.222	0.285	0.099
Lipofuscin bodies	4.233	1.522, 18.265	0.040	0.260
Atypically dividing cells	3.855	2.212, 26.544	0.030	0.243
Main effect – phenol treatment				
Cell count	9.024	5, 12	<0.001,	0.790
Spherical shape cells	15.748	5, 12	<0.001,	0.868
Hypertrophied cells	4.829	5, 12	0.012	0.668
Monster cells	6.167	5, 12	0.005	0.720
Lipofuscin bodies	0.854	5, 12	0.538	0.262
Atypically dividing cells	5.591	5, 12	0.007	0.700

Data were checked for homogeneity of variation and sphericity. As our data violated the assumption of sphericity, a repeated measures ANOVA with a Greenhous-Geisser correction was used.
F, Fisher F-test; df, degree of freedom.
Statistical significance at P < 0.05 is highlighted by pink color.

Table S2. Two-way ANOVA: Test of pairwise differences (factor: phenol treatment) for cell count, spherical shape cells, hypertrophied cells, monster cells, lipofuscin bodies and atypically dividing cells of *Euglena gracilis.*

Group (I)	Group (J)	Mean Dif. (I-J)	Std. Error	Sig.	95% CI for Difference	
					Lower Bound	Upper Bound
Cell count × 10^5/ml						
Control	3.81 mM	2.159	2.152	1.000	-5.694	10.012
	4.23 mM	5.778	2.152	0.298	-2.075	13.631
	4.65 mM	9.194	2.152	0.016	1.341	17.047
	5.07 mM	8.173	2.152	0.038	0.320	16.026
	5.49 mM	12.259	2.152	0.001	4.406	20.112
Spherical shape cells, %						
Control	3.81 mM	-28.828	5.341	0.002	-48.316	-9.339
	4.23 mM	-30611	5.341	0.001	-50.100	-11.123
	4.65 mM	-34.261	5.341	<0.001	-53.750	-14.773
	5.07 mM	-37.044	5.341	<0.001	-56.533	-17.556
	5.49 mM	-42.678	5.341	<0.001	-62.166	-23.189
Hypertrophied cells, %						
Control	3.81 mM	-1.317	3.149	1.000	-12.808	10.174
	4.23 mM	-2.894	3.149	1.000	-14.386	8.597
	4.65 mM	-3.539	3.149	1.000	-15.030	7.952
	5.07 mM	-9.556	3.149	0.156	-21.047	1.936
	5.49 mM	-12.339	3.149	0.031	-23.830	-0.848
Monster cells, %						
Control	3.81 mM	-0.100	0.482	0.839	-1.151	0.951
	4.23 mM	-0.117	0.482	0.813	-1.168	0.934
	4.65 mM	-0.661	0.482	0.196	-1.712	0.390
	5.07 mM	-0.956	0.482	0.071	-2.007	0.096
	5.49 mM	-2.228	0.482	<0.001	-3.279	-1.177
Lipofuscin bodies, %						
Control	3.81 mM	-1.700	0.971	1.000	-5.244	1.844
	4.23 mM	-0.906	0.971	1.000	-4.450	2.639
	4.65 mM	-1.128	0.971	1.000	-4.672	2.417
	5.07 mM	-1.133	0.971	1.000	-4.678	2.411
	5.49 mM	-0.211	0.971	1.000	-3.756	3.333
Atypically dividing cells, %						
Control	3.81 mM	-0.100	0.822	1.000	-3.101	2.901
	4.23 mM	-0.511	0.822	1.000	-3.512	2.490
	4.65 mM	-1.617	0.822	1.000	-4.617	1.384
	5.07 mM	-2.339	0.822	0.222	-5.340	0.662
	5.49 mM	-3.428	0.822	0.020	-6.429	-0.427

CI, confidence interval.
Based on estimated marginal means.
Statistical significance at $P < 0.05$ is highlighted by pink color.
Adjustment for multiple comparisons: Bonferroni.

Table S3. Two-way ANOVA: Test of pairwise differences (factor: time) for cell count, spherical shape cells, hypertrophied cells, monster cells, lipofuscin bodies and atypically dividing cells of *Euglena gracilis*.

Time (I)	Time (J)	Mean Dif. (I-J)	Std. Error	Sig.	95% CI for Difference	
					Lower Bound	Upper Bound
Cell count × 10^5/ml						
1h	24h	-0.037	0.075	1.000	-0.309	0.236
	3d	-0.812	0.284	0.217	-1.849	0.225
	7d	-7.622	1.256	<0.001	-12.205	-3.039
	10d	-12.406	1.609	<0.001	-18.277	-6.535
	14d	-14.823	1.995	<0.001	-22.103	-7.543
Spherical shape cells, %						
1h	24h	-19.794	12.399	1.000	-65.037	25.448
	3d	-8.967	4.093	0.734	-23.903	5.969
	7d	19.222	5.392	0.058	-0.454	38.898
	10d	18.822	6.008	0.130	-3.099	40.743
	14d	2.128	9.145	1.000	-31.242	35.497
Hypertrophied cells, %						
1h	24h	-2.439	0.598	0.023	-4.621	-0.257
	3d	-6.011	2.650	0.638	-15.680	3.658
	7d	-0.967	0.448	0.782	-2.603	0.670
	10d	-7.811	1.475	0.003	-13.194	-2.428
	14d	-10.517	4.165	0.400	-25.715	4.681
Monster cells, %						
1h	24h	0.000	0.000	.	0.000	0.000
	3d	-0.722	0.348	0.060	-1.481	0.037
	7d	-1.233	0.540	0.041	-2.410	-0.056
	10d	-0.817	0.464	0.104	-1.827	0.194
	14d	-1.289	0.846	0.153	-3.131	0.554
Lipofuscin bodies, %						
1h	24h	-0.928	0.571	1.000	-3.013	1,157
	3d	0.000	0.000	.	0.000	0.000
	7d	0.000	0.000	.	0.000	0.000
	10d	-1.033	0.577	1.000	-3.139	1.072
	14d	-3.117	1.246	0.417	-7.662	1.428
Atypically dividing cells, %						
1h	24h	0.000	0.000	.	0.000	0.000
	3d	-0.650	0.306	0.827	-1.767	0.467
	7d	-2.861	0.624	0.009	-5.136	-0.586
	10d	-3.083	1.256	0.455	-7.668	1.501
	14d	-1.400	0.850	1.000	-4.501	1.701

CI, confidence interval; h, hour; d, day(s).

Based on estimated marginal means.

Statistical significance at $P < 0.05$ is highlighted by pink color.

Adjustment for multiple comparisons: Bonferroni.
